# Hydrogels in Heritage Conservation: A Comparative Evaluation on Composite Objects

**DOI:** 10.3390/gels11100828

**Published:** 2025-10-15

**Authors:** Stavroula Rapti, Stamatis Boyatzis, Athanasios Velios, Shayne Rivers, Anastasia Pournou

**Affiliations:** 1 Department of Conservation of Antiquities and Works of Art, University of West Attica, Aegaleo, 12243 Athens, Greece; sboyatzis@uniwa.gr (S.B.); pournoua@uniwa.gr (A.P.); 2 The Engine House, English Heritage, Swindon SN2 2EH, UK; athanasios.velios@english-heritage.org.uk; 3 West Dean College of Arts and Conservation, Chichester PO18 0QZ, UK; riverscons@gmail.com

**Keywords:** conservation, wood, textile, chelators, agarose, extra-dry Nanorestore^®^ gel, cultural heritage

## Abstract

Ethnographic collections often incorporate composite objects consisting of various materials, including wood, textiles and metals. These objects are vulnerable to deterioration when iron fastenings corrode under humid environments, and their removal is therefore essential for the long-term preservation of artifacts. This study investigates the efficacy of the chelating agents Desferrioxamine B (DFO-B) and ethylenediaminetetraacetic acid (EDTA), applied in different gel formulations, in cleaning wooden and textile mock-ups stained with iron corrosion products. Three gel types were explored: xanthan gum, agarose and Nanorestore extra-dry gel with medium water retention (nano-MWR). The results indicated that xanthan gum exhibited the highest cleaning effectiveness but posed risks of residue deposition and surface damage due to the required clearance process. Agarose and nano-MWR gels proved to be less effective but showed potential for achieving high chelator efficacy with repeated applications. Agarose enhanced the chelators’ efficacy on textiles, while nano-MWR gel performed better on even wooden surfaces. No chemical damage was detected for either substrate across gel applications. The study concludes that a single gel formulation does not achieve equivalent cleaning efficacy on the two substrates of composite objects with a defined number of applications. Agarose in a semi-rigid state enhances the efficacy of textile treatment and may achieve comparable results on wood after repeated applications. Alternatively, a combined approach using agarose for textiles and nano-MWR gel for wood may optimize chelator performance on composite artifacts.

## 1. Introduction

Ethnographic collections often contain composite objects, such as furniture, household utensils, religious artifacts, toys, musical instruments and books, that are made of a combination of materials like wood, metal and textiles. Iron fastenings, including nails, screws and hinges, are frequently found in these objects but are prone to corrosion when exposed to environments with a relative humidity above 65%. The resulting iron oxides and hydroxy-oxides, such as goethite, hematite, lepidocrocite, akageneite, maghemite and magnetite [1,2,3,4], can severely damage the organic components like wood and textiles. Therefore, the removal of these corrosion products is critical to preserving the integrity of such artifacts [3,5,6].

Cleaning is an important aspect of conservation, as it is an invasive and irreversible process. Therefore, a cleaning methodology must involve the careful selection of suitable materials and techniques to remove harmful iron ions while minimizing any alteration to the physicochemical properties of the artifact’s original organic substrates.

The removal of iron ions has been extensively studied across various substrates, including paintings, paper, textiles, metals, ceramics, stone and waterlogged wood [7,8,9,10,11,12,13,14,15,16]. EDTA has been widely used in the conservation of cultural heritage objects; however, there is a notable lack of research on the application of chelating agents to dry composite objects. Moreover, conventional chelators like EDTA do not meet current environmental standards, highlighting the need for more eco-friendly alternatives [17]. Siderophores, natural iron chelators produced by microorganisms and plants in iron-deficient conditions, represent a promising “green” solution [18,19]. These hexadentate ligands have a high affinity for ferric ions, reduce environmental risks and prevent harmful Fenton reactions without generating hydroxyl radicals [20,21,22]. Desferrioxamine B (DFO-B), a siderophore, has proven effective in removing ferric compounds from cultural heritage materials, including paper [23,24], waterlogged wood [25,26,27], metals [28,29], and dry wood and textiles [30,31].

Both conventional chelators and siderophores are water-soluble substances, requiring some degree of water exposure for application, typically through immersion. However, this presents challenges when cleaning composite objects made of wood and textiles.

In textile conservation, immersion in aqueous baths, commonly referred to as “wet cleaning,” is a standard method for removing stains, dirt and soiling. In contrast, immersing wooden objects in aqueous baths poses significant risks. Wood is both hygroscopic and anisotropic and thus reacts unevenly to water exposure. This can lead to swelling, distortion or warping along the tangential, radial and axial directions. Additionally, immersion may increase the penetration of corrosion products into wood, loosen joints or cause cracking. The drying process that follows can also result in further shrinkage and structural damage.

In some cases, dismantling a composite object to treat each material separately may be considered. However, this approach carries considerable risks and is often unsafe or impractical, potentially compromising the object’s structural integrity [11,32]. Conservation ethics emphasizes “minimal intervention,” making dismantling a last-resort option. Instead, non-invasive or low-impact treatment methods are preferred to preserve the object’s original form and condition [33].

Thus, for composite objects that include wood, conventional cleaning methods pose significant challenges, as treatments effective for one material may be harmful to another [11]. Cleaning methodologies for dry wooden substrates must be compatible with wood’s hygroscopic and anisotropic nature. Therefore, cleaning should be performed in a controlled and selective manner, using materials that limit water release while maintaining effective cleaning action. Water release has been widely studied in the field of conservation, particularly with regard to the rate of diffusion of water or liquids from various polymeric gel networks into substrates [34,35,36,37,38,39].

Gel formulations meet these criteria and offer a viable solution for composite objects, particularly those combining wood and textiles. Thickening agents such as polyacrylic acid, xanthan gum and cellulose ethers, first introduced by Wolbers in the mid-1980s [40], have been widely adopted for this purpose. However, these gels require thorough rinsing to avoid leaving residues on porous surfaces due to the weak cohesion of their polymer networks [41,42].

To minimize residues, rigid physical gels like agarose, agar and low-acyl (LA) gellan gum and chemical gels like Poly(vinyl alcohol)-borax gels (PVA-B), which recently were combined with agarose, have been widely used with successful results across various substrates, including stone, textiles, paper, ceramics and paintings [16,43,44,45,46,47,48,49,50]. More recently, “chemical hydrogels” with covalently cross-linked polymer networks have been developed, such as Nanorestore^®^ Extra Dry with medium water retention (MWR) or Max Dry with high water retention (HWR), consisting of poly(vinyl pyrrolidone) (PVP) chains embedded in a poly(2-hydroxyethyl methacrylate) (pHEMA) network [51]. These gels offer strong cohesive forces, mechanical and pH stability, and high retention of cleaning solutions, making them particularly well-suited for water-sensitive substrates like wood [41,42,52,53]. Transparent and residue-free, these hydrogels have proven to be effective for cleaning a wide range of materials, including paintings, metals, textiles and varnished furniture [54,55,56,57,58,59,60,61,62].

This study aimed to investigate the efficacy of chelating agents applied in different gel formulations on two distinct substrates (wood and textiles) within a predefined application time. The primary objective was to identify the most effective gel formulation for each substrate and to recommend a suitable formulation for composite objects composed of both materials. Additionally, the study sought to determine any chemical impact on the substrates resulting from the cleaning process.

For this purpose, Nanorestore^®^ MWR as a suitable gel for water-sensitive materials was tested alongside agarose and xanthan gum gels on wooden and textile mock-ups stained with iron corrosion products. The selection of these gels was based on several criteria: their availability and compatibility with the substrates, as well as their transparency and purity. Low-acyl gellan gum (LA) met the requirements for compatibility, transparency and purity; however, it was not included in the study, due to its availability only in large quantities, which exceeded the practical needs of the project. Compatibility was prioritized to avoid chemical alteration of the wooden and textile substrates. PVA–borax gels, although meeting most of the selection criteria and having been successfully employed in combination with chelators [16], were excluded due to their requirement of alkaline conditions (pH > 8), which carry a risk of alkaline hydrolysis. Additionally, these gels become viscous liquids at pH levels below 7–7.5 [46], limiting their applicability under neutral or slightly acidic conditions. Gel purity was also considered crucial to reduce experimental variability during the cleaning tests, while optical transparency enabled real-time visual monitoring of cleaning and complexation processes. This is particularly relevant given that iron complexes show distinct colors (e.g., pale yellow for EDTA–Fe [63] and orange-red for DFO–B-Fe [64]). For these reasons, agarose was selected over agar, despite the latter’s lower cost and its wide use in conservation practice [36,43,47,48,60,65,66] due to its higher purity and superior clarity. Xanthan gum was chosen as the thickener owing to its demonstrated applicability across a range of cultural heritage materials [67,68,69].

Two chelating agents, Desferrioxamine B (a siderophore) and EDTA (a synthetic chelator), were assessed for their cleaning effectiveness using Energy-Dispersive X-Ray Spectroscopy (EDS) and colorimetric analysis. To evaluate possible alterations to the substrates and detect any surface gel residues post-cleaning, Attenuated Total Reflection–Fourier Transform Infrared Spectroscopy (ATR-FTIR) and Scanning Electron Microscopy (SEM) were employed.

## 2. Results and Discussion

### 2.1. Cleaning Efficacy

#### 2.1.1. Energy-Dispersive X-Ray Spectroscopy (EDS)

**Figure 1:**
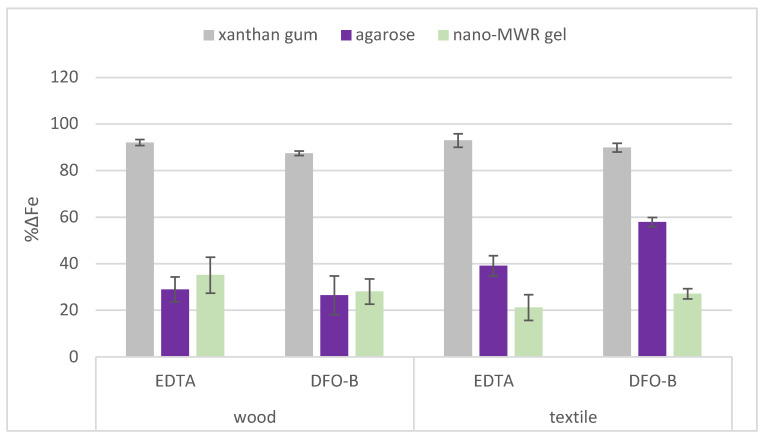
Comparative graph of the average cleaning efficacy (%ΔFe) values calculated from the iron weight (%wt) detected by EDS for the six replicates of wooden and six replicates of textile mock-ups after application of EDTA and DFO-B with xanthan gum, agarose and nano-MWR gels.

The cleaning efficacy was assessed by %ΔFe (see Section 4.3.1), which represented the difference in the amount of iron compounds present on substrates before and after the cleaning treatments, where higher values indicate greater removal of iron. The EDS results showed that the %ΔFe increased with repeated applications and that the efficacy of the chelators varied depending on both the substrate and the type of gel, as shown in Figure 1.

Specifically, when EDTA was applied with xanthan gum, the highest %ΔFe was reached on textile mock-ups (93.0) after eight applications, while slightly lower values were recorded on wooden mock-ups (92.1). Similarly, DFO-B applied with xanthan gum resulted in a higher %ΔFe on textiles (89.9) compared to wood (87.5).

The application of EDTA with agarose in a semi-rigid state resulted in an %ΔFe of 39.1 on textile mock-ups, with a noticeably lower value of 29% on wood. Similarly, DFO-B combined with agarose showed considerably higher efficacy on textiles (%ΔFe: 57.9) compared to wood (%ΔFe: 26.4).

However, the efficacy of chelators when applied with nano-MWR gel was higher on wooden mock-ups compared to textiles. Specifically, EDTA reached %ΔFe (35.1) after eight applications on wooden mock-ups, with a lower efficacy on textile mock-ups (21.1). Likewise, DFO-B reached an %ΔFe of 27.1 on wooden mock-ups and a slightly lower value on textile mock-ups (21.1).

By comparing the three different gel formulations, it becomes apparent that both chelators exhibited greater efficacy when applied with xanthan gum gel on both substrates as opposed to agarose or nano-MWR gel (Figure 1). This improved performance is likely attributed to the mechanical action involved in the clearance process using a cotton swab, which enhanced the removal of corrosion products, as well as the gel itself.

More specifically, xanthan’s anionic structure [70,71] may have contributed to more effective chelation. Although it was applied via a barrier material, the gel was able to penetrate through the micro-scale interstices of the barrier due to its semi-permeability. This partial contact allows the anionic nature of xanthan gum to remain relevant, as it may still influence local chelation dynamics at the interface. Additionally, its flexible texture allowed for closer contact with the substrate’s surface morphology and may have facilitated greater release of chelators. Therefore, the complexation action was probably promoted, since the rate of extraction depends on the diffusion rate of the solution within the substrate [12]. Nonetheless, no definite conclusions could be drawn regarding the individual effectiveness of each chelator in extracting ferric compounds when applied with xanthan gum due to the synergistic role of the clearance process.

Regarding the rigid gels (agarose and nano-MWR), although agarose is known to enhance chelation through its polymeric network [48,72], no published evidence has yet demonstrated a similar chelating effect for nano-MWR. The performance of both rigid gels in enhancing the efficacy of chelators after eight applications was substrate-dependent. Although the overall effect was moderate, an upward trend was observed, suggesting that repeated applications could lead to increased effectiveness. In particular, the chelators performed better on textile mock-ups when applied with agarose, likely due to its semi-rigid state during application, which allowed for close contact with the uneven surface of the textiles. This close contact, combined with the higher release capacity of the chelators’ solution, enhanced complexation and consequently facilitated the iron removal. These findings are consistent with the results reported by Cuvillier et al. [28], where DFO-B and EDTA showed greater efficacy when applied using hot agar gels versus more rigid forms of agar gels, particularly on uneven surfaces of corroded metallic objects. In contrast, the efficacy of chelators on wooden mock-ups was lower than on textiles, despite the wood’s flat and even surface and the gel’s good contact with it. This reduced performance may be related to the semi-rigid state of the gel during application, which likely caused an immediate release of a small amount of chelator. Due to the elevated temperature of the gel, it is likely that the chelators’ mobility was accelerated, resulting in higher formation of soluble complexes. These factors, along with the prolonged application time, may have contributed to the overall lower chelation efficacy observed on wooden substrates. Since complexation was visually observed significantly earlier (approximately 15 min) than the defined application time, it can be assumed that the diluted chelator complexes remained on the surface. This suggests that the complexes were not fully absorbed into the agarose gel, likely due to insufficient adhesion to the substrate or an inadequate clearance process. Previous studies have demonstrated that application time has a synergistic role in achieving optimal results. In particular, repeated short applications have been shown to be more effective in removing ferric compounds than fewer prolonged applications [28]. Another possible explanation for the higher %ΔFe values observed on textiles could be related to the different quantities of iron compounds formed during the artificial staining process. Indeed, almost twice as much iron was deposited on the wooden substrates compared to the textiles. Although the same type of ferric compounds formed on both substrates, their relative proportions differed. Thus, the better performance of DFO-B on textiles may be attributed to its preferential affinity for maghemite (γ-Fe_2_O_3_), which was present in a higher percentage on the textile substrate.

On the other hand, nano-MWR gel demonstrated a higher efficacy when chelators were applied to the wooden mock-ups. This is probably due to its highly retentive network, which enhanced adhesion to the flat and even wooden surfaces. The gel required gentle force to be peeled off, suggesting strong surface interaction, despite its relatively low chelator release and the unknown quantity of chelators it had absorbed during immersion, an important factor in complexation efficacy [7,21,73,74]. Thus, it may have more effectively and homogeneously absorbed the solubilized iron compounds and their resulting complexes compared to agarose. The consistent performance of nano-MWR gel has also been reported by other researchers [62]. In contrast, its application on textile mock-ups resulted in lower chelator efficacy. This was likely due to the textiles’ uneven surfaces, which limited contact with the gel and led to poor and uneven substrate wetting—conditions that do not favor efficient complexation. Similar limitations of nano-MWR gel on irregular surfaces, such as corroded metal, have been previously reported [60]. However, there are case studies in which nano-MWR gel was effectively used on textiles, particularly in treatments where only swelling of the adhesive was needed for successful separation [56].

#### 2.1.2. Colorimetry

The total color difference (ΔE*) represented the change in substrate color resulting from the cleaning treatments, with higher values indicating greater removal of ferric compounds. Compared to the ΔE* values for untreated stained mock-ups, those for the mock-ups treated with chelators in gels progressively increased with repeated applications. Additionally, the extent of the color change varied depending on the type of gel formulation.

**Figure 2:**
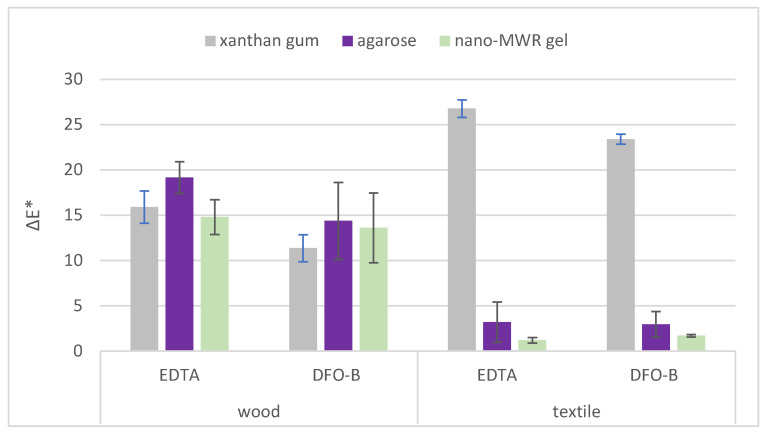
Comparative graph of the average ΔE* values for the six replicates of wooden and six replicates of textile mock-ups after the application of EDTA and DFO B with xanthan gum, agarose and nano-MWR.

Specifically, ΔE* values for both EDTA and DFO-B were considerably higher on textile mock-ups when applied with xanthan gum compared to wooden mock-ups, even though the EDS results for both substrates were relatively similar. For example, after xanthan gum application, ΔΕ* values for EDTA reached 26.78 on textiles and 15.92 on wood, while those for DFO-B reached 23.40 and 11.37, respectively (Figure 2).

The ΔE* values for both chelators applied with agarose gel did not align with the results obtained by EDS. Colorimetry values were higher when chelators were applied to wooden rather than textile mock-ups. For EDTA, the ΔE* reached 19.20 on wood compared to just 3.20 on textiles, whereas DFO-B showed a ΔΕ* of 14.40 on wood and 2.96 on textiles (Figure 2).

Regarding the application with nano-MWR gel, the ΔE* for EDTA on wooden mock-ups was 14.80 and that on textiles was 1.20, and the values for DFO-B were 13.60 and 1.70, respectively (Figure 2).

**Figure 3:**
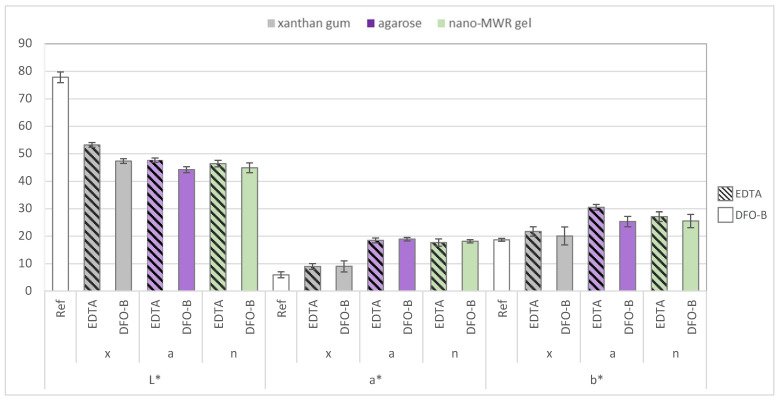
Comparative graph of L*, a*, b* average values for ten reference unstained wooden mock-ups and six replicates of wooden mock-ups after treatment with chelators. x, a and n correspond to xanthan gum, agarose and nano-MWR gel, respectively, with which EDTA and DFO-B were applied.

To summarize, the colorimetry results were not fully consistent with those obtained by EDS in evaluating cleaning efficacy for the wooden mock-ups. Among the tested gel formulations, the application of chelators, especially EDTA, resulted in the highest ΔE* on wood when applied with agarose. Although ΔE* is a widely used metric in conservation, [75,76,77], it is often necessary to consider the values of color coordinates (L*, a*, b*) to obtain more reliable and comparable data [47,78]. In this study, the L*, a*, b* coordinates of the chelator applications using xanthan gum were found to be closest to those of the reference (unstained) wooden mock-ups, thus indicating better cleaning efficacy (Figure 3).

### 2.2. Presence of Residues

**Figure 4:**
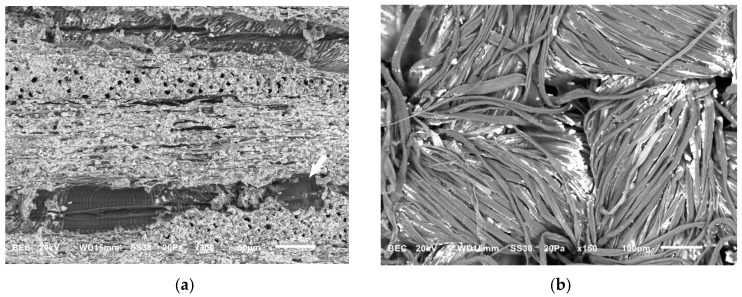
SEM micrographs of (**a**) wooden mock-up, where agarose residue is observed in the vessel lumen (arrow), and (**b**) textile showing agarose residues at the interlacing points of threads (arrows).

Both types of mock-ups were examined using Scanning Electron Microscopy (SEM) to assess the presence of any residues following the application of chelators with different gel formulations. Agarose residues were rarely observed (in one of the six mock-ups) within the lumens of vessels of the wood (Figure 4a) and at the interlacing points of the textile fibers (Figure 4b), possibly due to the semi-rigid state during application. In contrast, no visible residues were detected on either substrate following the use of chelators with the nano-MWR gel.

**Figure 5:**
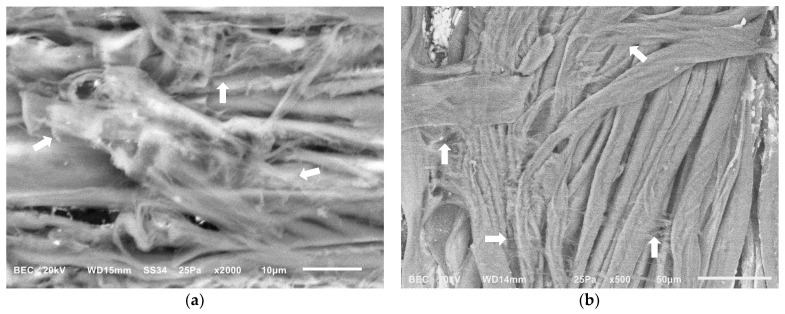
SEM micrographs, where residues of xanthan gum in the form of spider’s webs covering the cells were detected on the surfaces of (**a**) wooden mock-ups (arrows) and (**b**) textile mock-ups prior to the clearance process after the application of chelators (arrows).

**Figure 6:**
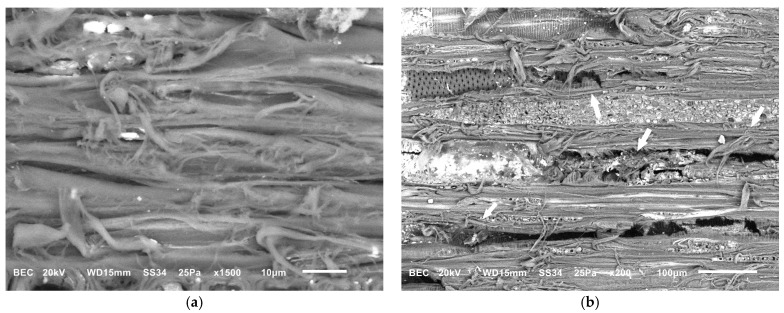
SEM micrographs of wooden mock-ups: (**a**) fraying and partial removal of fibers can be observed, along with (**b**) distortion of fragmented elements of vessels (arrows).

The Japanese paper used as a barrier for xanthan gum application was semi-permeable and had a low mechanical strength. While it enabled controlled chelator diffusion, gel migration occurred through the narrow interstices of the non-woven threads, and the barrier frequently failed during removal, presenting partial rupture, resulting in gel deposition on the surface (Figure 5a,b). Consequently, to prevent residue presence following the application of chelators with xanthan gum, an intensive clearance process was required. In contrast, the rigid gels, applied in direct contact, maintained their structural integrity and left negligible amounts of residue, eliminating the need for post-treatment clearance. This procedure led to significant mechanical alteration of wood cells. These effects were attributed to the repeated abrasion caused by the use of swabs during the clearance process (Figure 6a,b).

**Figure 7:**
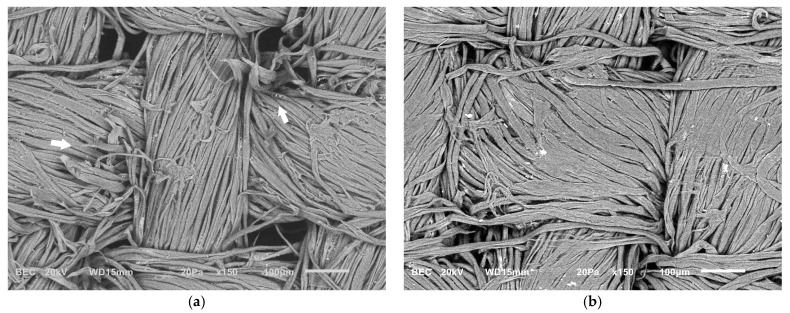
SEM micrographs of textile mock-ups showing (**a**) broken fibers and weave distortion (arrows) and (**b**) flattening of fibers at the apex of the thread curve.

Similarly, in the textile mock-ups, SEM revealed significant weave distortion and deformation accompanied by fraying and partial fiber detachment (Figure 7a). Additionally, changes in fiber morphology were observed, with many fibers appearing flattened at the apex of the thread curve in both the weft and warp (Figure 7b).

In contrast, no structural alterations were detected in wooden or textile mock-ups following the application of chelators with agarose or nano-MWR gel. SEM imaging confirmed that the cellular structure of the wood remained intact and that the weave of the textiles was undisturbed.

### 2.3. Cleaning Impact on Substrates

The impact of the cleaning treatments on both substrates was examined through ATR-FTIR spectra. Assessment of the wood and cotton conditions after the cleaning treatments was performed by comparing spectra after all cleaning treatments with reference spectra for maple wood and cotton recorded before staining and after staining. Infrared bands and assignments for the reference maple wood and cotton are listed in Table 1.

**Figure 8:**
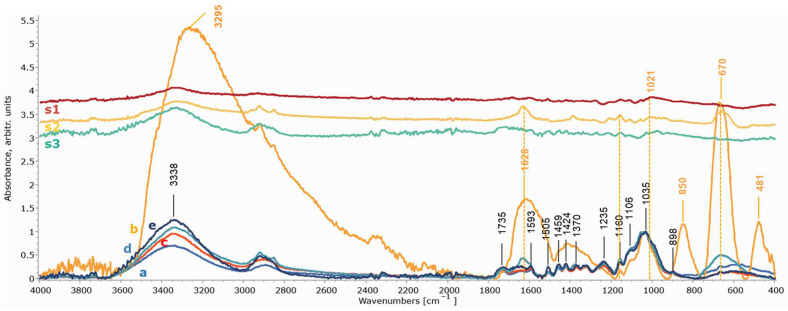
ATR-FTIR spectra of wooden mock-ups after eight consecutive applications of EDTA with xanthan gum (c), agarose (d) and nano-MWR gel (e) in comparison to reference spectra of maple wood (a) and stained maple before cleaning treatments (b). Spectra (s1), (s2) and (s3) correspond to the subtraction between the EDTA applications with xanthan gum, agarose and nano-MWR and the reference maple mock-up, respectively. Spectra were normalized at 1035 cm^−1^.

**Figure 9:**
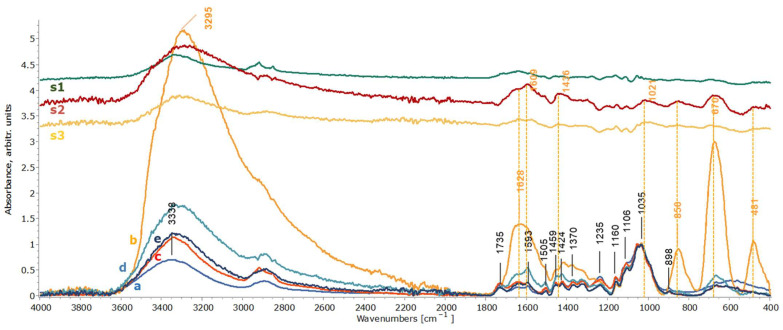
ATR-FTIR spectra of wooden mock-ups after eight applications of DFO-B with xanthan gum (c), agarose (d) and nano-MWR gel (e) in comparison to reference spectra of maple wood (a) and before cleaning treatments (b). Spectra (s1), (s2) and (s3) correspond to the subtraction between the DFO-B applications with xanthan gum, agarose and nano-MWR and the reference maple mock-up, respectively. Spectra were normalized at 1035 cm^−1^.

**Figure 10:**
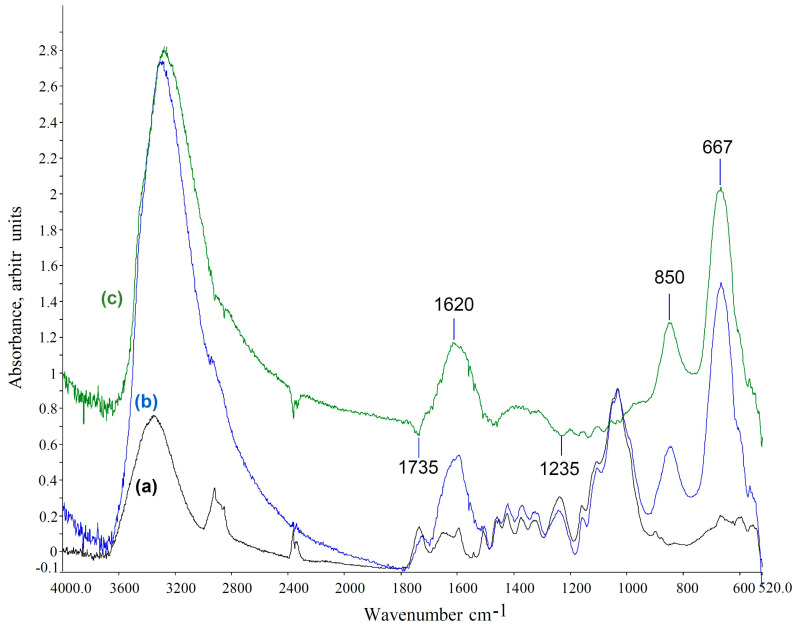
ATR-FTIR spectra of reference mock-up (a) and artificially stained maple mock-up (b). The subtraction spectrum (c) is between the (b) and (a) spectra.

The infrared spectra of the artificially stained wooden mock-ups before the cleaning treatments did not clearly show the expected cellulose and lignin vibrations of maple wood due to the produced layer of iron oxides/hydroxy-oxides that creates a masking film on the wood surface, which can be seen by comparing Figure 8b and Figure 9b with the spectra acquired from the reference (fresh) maple wood (Figure 8a and Figure 9a). The most prominent bands are attributed to akageneite (850 and 481 cm^−1^) and to maghemite (670 cm^−1^), while the ones at 1628 cm^−1^ and 3295 cm^−1^ are associated with OH of akageneite (iron hydroxy-oxide) and bonded water [2,91,92,93]. However, the spectra obtained for the cleaned wooden mock-ups with both chelators applied in the three gel formulations clearly show, especially in the case of xanthan gum, all bands associated with maple wood vibrations, i.e., hemicellulose/pectins are clearly detected at 1735 cm^−1^ and 1235 cm^−1^; cellulose is detected at 898 cm^−1^ and holocellulose at 1370 cm^−1^, 1160 cm^−1^ and 1035 cm^−1^; as well as lignin at 1593 cm^−1^, 1505 cm^−1^ and 1424 cm^−1^. Bands at 1459 and 1424 cm^−1^ are assigned to both lignin and cellulose [79,80,81,82,83,84]. The maple hemicelluloses, detected at 1735 and 1235 cm^−1^ in the mock-ups’ spectra obtained after the cleaning treatments, the most vulnerable components of the wood, were slightly decreased, indicating a small removal (Figure 8c–e and Figure 9c–e). Given that such changes were evident in mock-ups after the selected staining process (Figure 10), these observations did not imply further noticeable chemical alteration owing to the cleaning procedure concerning the C=O of xylan hemicellulose and C-H deformation of carbohydrates.

Nevertheless, the ATR-FTIR analysis also highlighted that the type of gel formulation influenced the efficacy of the chelators, confirming the EDS results (Figure 8 and Figure 9). The varying effectiveness of the chelators in removing iron oxides/hydroxy-oxides was clearly illustrated by the gradual reappearance of the above-mentioned bands of maple wood and the additional reductions in the akageneite and maghemite peaks at 3295, 1628, 850 and 670 cm^−1^. No detectable amounts of iron oxides/hydroxy-oxides were evident after cleaning with xanthan gum and nano-MWR gel, in contrast to the application of agarose (Figure 8b–e and Figure 9b–e). As shown by the subtraction spectra of mock-ups treated with both chelators with agarose (Figure 8s2 and Figure 9s2), small amounts of iron oxides/hydroxy-oxides were evident (more after DFO-B application) due to remaining akageneite (at 1628, 1609, 850 and 481 cm^−1^) and maghemite (at 670 cm^−1^) and probably traces of lepidocrocite (at 1436, 1160 and 1020/1021 cm^−1^) [2,91,92,93]. Additionally, there was no clear evidence of gel residues in the application of both chelators, as shown by the subtraction spectra (Figure 8s1–s3 and Figure 9s1–s3).

**Figure 11:**
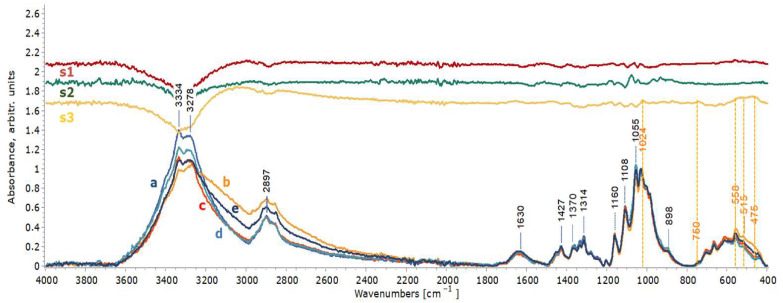
ATR-FTIR spectra of textile mock-ups after eight applications of EDTA with xanthan gum (c), agarose (d) and nano-MWR (e) in comparison to reference spectra for cotton (a) and stained cotton mock-ups before cleaning (b). Spectra were normalized at 1032 cm^−1^. Spectra (s1), (s2) and (s3) correspond to the subtraction between the EDTA applications with xanthan gum, agarose and nano-MWR and the reference maple mock-up, respectively.

**Figure 12:**
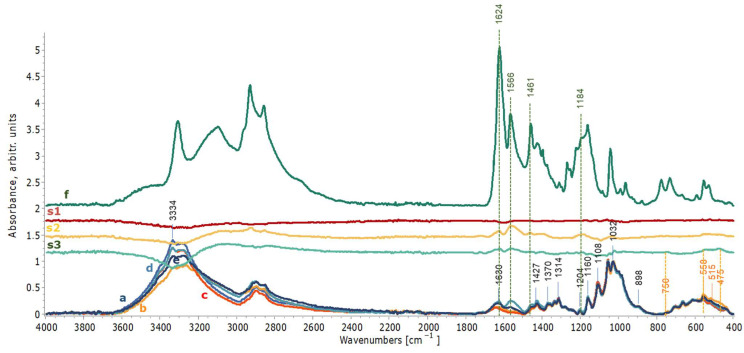
ATR-FTIR spectra of textile mock-ups after eight applications of DFO-B with xanthan gum (c), agarose (d) and nano-MWR (e) in comparison to reference spectra for cotton (a) and stained mock-ups before cleaning treatments (b). Spectra were normalized at 1032 cm^−1^. Spectrum (f) was obtained from desferal reference material. s1, s2 and s3 correspond to the subtractions between DFO-B application with xanthan gum, agarose and nano-MWR and the reference maple mock-up, respectively.

ATR-FTIR spectra were also obtained to assess the potential impact of cleaning applications on the textile mock-ups. In the case of textiles, the produced iron oxides/hydroxy-oxides did not create a masking film on the cotton vibrations, probably due to the lower quantity of iron compounds compared to wood and due to their penetration within the textile fibers opposed to the concentrated compounds produced on the surface of the wooden mock-ups. These may have fallen below the detection threshold of ATR-FTIR. However, the spectra for each chelator applied by the three gels were compared. In particular, spectra obtained after EDTA and DFO-B applications with the three gels on stained textiles presented no changes in any of the characteristic vibrations of cotton (1427, 1370, 1314, 1160, 1108, 1032 and 898 cm^−1^) (Figure 11a–e and Figure 12a–e). Minor bands at 1024, 750, 558, 515 and 475 cm^−1^ that were present in the stained mock-ups were assigned to iron compounds, such as maghemite, akageneite and lepidocrocite, which were lower but still evident after the cleaning treatments with nano-MWR, confirming the EDS results (Figure 11e,s3 and Figure 12e,s3) [2,92]. No detected residues of xanthan gum or agarose were evident (Figure 11s1,s2 and Figure 12s1,s2).

As shown in Figure 12, in spectra (d) and (e) for mock-ups treated with DFO-B with agarose and nano-MWR gel formulations and their subtraction spectra (s2 and s3), peaks at 1624 and 1566 cm^−1^ are probably attributable to the CO stretching of hydroxamate and CNH of secondary amides, respectively. These features suggest the presence of uncomplexed DFO-B residues. Additionally, the weak peaks at 1461, 1427 and 1184 cm^−1^ are attributed to CH3, CH and CN vibrations, respectively, referable also to DFO-B residues [94]. This implies inadequate clearance of these mock-ups. These results demonstrate that textiles, probably due to their uneven surfaces and their ability to easily absorb substances, need a great number of repeated clearance steps to achieve efficient removal of uncomplexed chelators or even diluted complexes, which may alter their long-term preservation. Time of application was also considered as a possible factor that enhanced this process. The subtraction spectra for mock-ups treated with DFO-B with xanthan gum did not present any DFO-B residues (Figure 12c,s1).

### 2.4. Managing Redistribution at the Cleaning Zone Perimeter

The stereoscopic examination of the surfaces of the wooden and textile mock-ups demonstrated that both chelators effectively complexed with iron corrosion products. However, complete removal of these corrosion products was not achieved, even after eight repeated applications.

**Figure 13:**
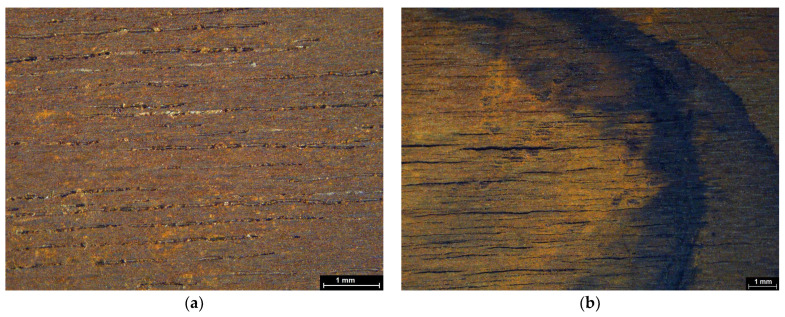
Maple mock-ups under stereomicroscope (**a**) before cleaning treatments and (**b**) after chelator application with xanthan gum. Scale bar: 1 mm.

**Figure 14:**
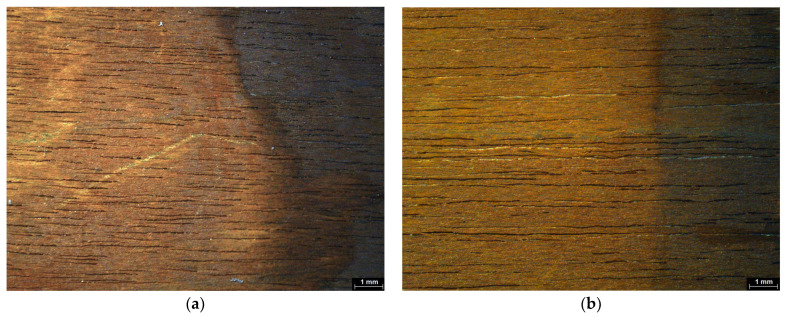
Comparison of maple mock-ups after chelator application using (**a**) agarose and (**b**) nano-MWR, observed under stereomicroscope. Scale bar: 1 mm.

All mock-ups treated with xanthan gum-based gels presented a visual accumulation of the solubilized iron compounds at the edges of the cleaning zone. This effect is probably due to the properties of xanthan gum, which tends to release a large amount of the chelator solution, and to the clearance process, which facilitated the redistribution of iron compounds toward the periphery (Figure 13a,b). In contrast, the applications using agarose and nano-MWR gels on wooden mock-ups resulted in more sharply defined cleaning zones, with the nano-MWR gel producing the most distinct boundaries between the cleaned and uncleaned areas without visible tidelines (Figure 14a,b). This effect can be attributed to the superior adhesion of the nano-MWR gel on the flat and uniform surface of wood, in contrast to the more irregular topographies of textile substrates. Other researchers have also reported the absence of tidelines in the areas on which nano-HWR was applied with solvents [62], due to its network ability to retain aqueous solutions or solvents. As for the less defined cleaning zones observed with agarose on wood, these were most likely due to its method of application in a warm semi-rigid state, which may have led to slightly increased chelator release upon application and less controlled cleaning boundaries.

**Figure 15:**
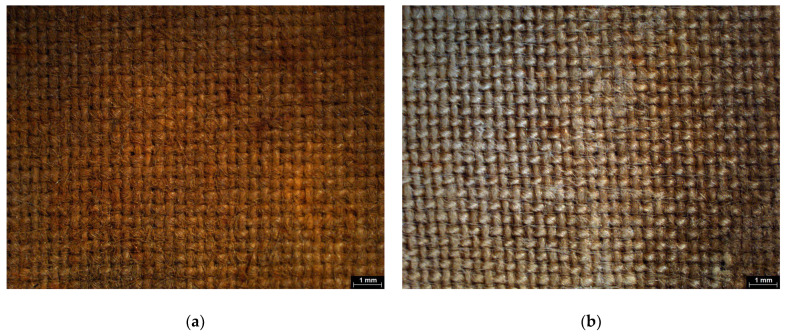
Cotton mock-ups under stereomicroscope (**a**) before cleaning treatments and (**b**) area after chelator application with xanthan gum (arrow). Scale bar: 1 mm.

On the textile mock-ups, the use of xanthan gum-based chelators led to considerable removal of iron compounds from the upper parts of the threads. Nonetheless, ferric compounds remained trapped at the interlacing points of adjacent weft and warp threads, likely due to the textile structure impeding chelator penetration and iron removal (Figure 15a,b).

**Figure 16:**
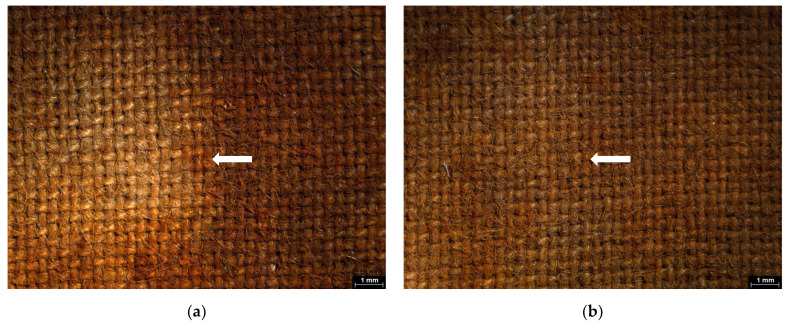
Areas of cotton mock-ups after chelator application using (**a**) agarose and (**b**) nano-MWR, observed under stereomicroscope (arrows). Scale bar: 1 mm.

In the case of the textile mock-ups, the cleaned areas were only slightly distinguishable from the untreated zones when chelators were applied using agarose gels. In contrast, the application of nano-MWR gel appeared to produce no visible cleaning effect. This result is likely due to the limited contact between the nano-MWR gel and the irregular surface of the textile substrate, which reduced the efficacy of the chelation process. Its inability to make contact on rough and irregular surfaces has been also reported for modern and contemporary art works [95]. In contrast, agarose, due to its semi-rigid form during application, offered a better contact and thereby facilitated a more effective interaction between the chelators and the iron corrosion products (Figure 16a,b).

### 2.5. Evaluation of Gel Formulations

**Figure 17:**
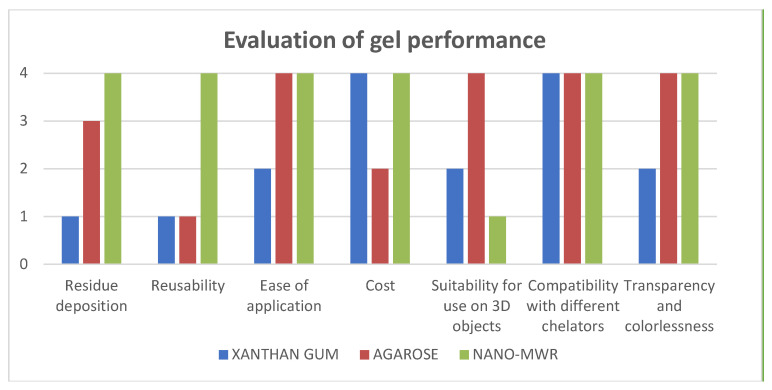
Comparative graph of the tested gels, xanthan gum, agarose and nano-MWR gels, concerning their properties and performance during conservation treatments with chelators. Grade 1 indicates minimal fulfillment of the criterion and 4 maximal fulfillment. The criteria for the assessments were (i) preventing residue deposition, (ii) reusability, (iii) ease of application (iv) cost, (v) suitability for use in 3D applications, (vi) compatibility with chelators and (vii) transparency and colorlessness.

The results obtained highlighted the importance of the gels’ formulations on the efficacy of the chelators. Therefore, their performance was comparatively evaluated based on the following criteria: (i) preventing residue deposition, (ii) reusability, (iii) ease of application, (iv) cost of purchasing the product, (v) suitability for use on two- and three-dimensional objects, (vi) compatibility with different chelators, and (vii) transparency and colorlessness (Figure 17). Based on this comparative assessment, it should be noted that although xanthan gum demonstrated high efficacy when combined with the tested chelators, in conservation practice it is considered inappropriate for uncoated surfaces. This is mainly due to the residues left post-application and the extensive clearance required, which can cause mechanical damage to both wood and textile substrates. As a result, it is not recommended for use on sensitive museum objects, and if used, it must be applied with great caution.

Since residue deposition is a primary concern among conservators, it is important to note that the nano-MWR gel demonstrated superior performance in this regard, followed by agarose in its semi-rigid state. Nano-MWR gel showed the best results in terms of ease of application and reusability. In this study, both nano-MWR and rigid agarose (prepared in a flat container) were reused only for rinsing purposes. Nano-MWR was effectively reused up to four times, while agarose was reused up to two times, after which it became brittle and lost its original cohesion. Although gel reuse during the clearance phase proved effective in this study, the reuse of gels for cleaning purposes should be carefully evaluated. It is essential to ensure that the gel network remains structurally intact after repeated use and that key cleaning parameters, such as the gel’s capacity to absorb cleaning solutions, remain consistent. Another advantage of the nano-MWR gel is its high ability to retain the chelator solution on hydroscopic wooden surfaces without significantly wetting them, while also producing well-defined cleaning boundaries with minimal tideline formation. However, although agarose ultimately formed a rigid gel at the relatively high concentration used (4% *w*/*v*), it was applied in a warm, semi-rigid state, in contrast to the cold nano-MWR gel. This difference in physical state at the time of application likely led to variations at the gel–substrate interface, resulting in distinct cleaning performance and, consequently, differences in overall chelation efficacy.

While nano-MWR gel enhanced the chelators’ efficacy on wooden substrates, it proved largely inefficient on the uneven surface topographies of the textile mock-ups, which did not allow adequate contact between the gel and the substrate. Additionally, nano-MWR gel is limited to application on flat two-dimensional surfaces or on slightly curved surfaces with the assistance of a weight to conform the shape, whereas agarose offers the potential for application on three-dimensional objects.

However, applying agarose in 3D applications requires the gel to be placed on the object as a viscous solution at a temperature a few degrees Celsius above its gelation point (45 °C). This allows the gel to conform to the surface shape while minimizing penetration into the substrate, particularly into textile fibers. This process, however, requires prior experience, which may compromise its ease of use.

Moreover, all gels tested were found to be compatible with the chelators investigated and permitted clear observation of color changes when complexation of iron ions occurred with the chelators. In the present study both nano-MWR gel and agarose demonstrated lower cleaning effectiveness compared to xanthan gum. However, their performance can be improved trough repeated applications, since neither gel caused any observable alteration to the substrates. However, in the case of museum objects, the number of applications should be tailored to the amount and depth of iron oxide/hydroxide penetration, while always considering the specific condition and preservation needs of the object.

All gels were compatible with the tested chelators, and due to their physicochemical properties and polymeric networks they enhanced chelator efficacy. However, chelator concentration plays a critical role in the chelation process. While xanthan gum and agarose gels were prepared with a known quantity of chelator, the amount absorbed by the nano-MWR gel remains unknown, complicating direct comparison. In conclusion, the findings of this study do not support the use of a single gel formulation with a selected chelator as a generally effective strategy for the optimal removal of ferric compounds from composite objects comprising both wood and textiles. However, agarose in a semi-rigid state was shown to enhance the efficacy of chelators on textiles and can achieve comparable results on wood after repeated applications. Its minimal residue deposition and lack of adverse effects on substrates make it a suitable choice, particularly for applications on 3D surfaces, commonly found in cultural heritage objects. Alternatively, a more ad hoc approach may be implemented using agarose on textile components and nano-MWR gel on flat or slightly curved wooden areas to maximize the performance of the chelators.

## 3. Conclusions

A sequence of repeated applications yielded satisfactory results, even though total removal of iron corrosion products was not achieved. However, the rigid gels were easily removed by simple peeling, avoiding more aggressive mechanical actions and limiting residue issues and subsequent clearance processes.

The removal of iron compounds during the application of chelators with xanthan gum was enhanced by the clearance process using a swab, which contributed significantly through a synergistic mechanical action. Consequently, the efficacy order of chelators in agarose and nano-MWR gels became more representative.

Because complexation is a multiparametric process, it is difficult to accurately compare the effectiveness of the gel formulations used. Nevertheless, it is clear that the application method, the gel properties and network, and the time of application all play an important role in the cleaning process, as they can either facilitate or inhibit chelation. Although both agarose and nano-MWR are rigid gels, their application methods (warm vs. cold) differ, and it is very likely that this influences chelator release, tideline formation and the gels’ pore structure. Additionally, agarose and xanthan gum, due to their chemical properties and polymeric structure, may contribute directly to the chelation mechanism. The superior performance of nano-MWR on wooden mock-ups, despite its absorbing an unknown amount of chelator, could be related to its network characteristics.

However, this study concludes that a single gel formulation with a selected chelator is not an effective method for removing ferric compounds from composite materials like wood and textiles. Semi-rigid agarose improves chelator effectiveness on textiles and can yield similar results on wood after multiple applications, due to its low residue levels and non-harmful effects on materials. For optimal results, a customized approach using agarose for textiles and nano-MWR gel for flat or slightly curved wooden surfaces may be more effective.

## 4. Materials and Methods

### 4.1. Mock-Up Preparation

To identify the most effective gel formulation for chelators to complex with iron corrosion products on composite objects comprising both wood and textiles, it was necessary to create separate mock-ups for each material.

**Figure 18:**
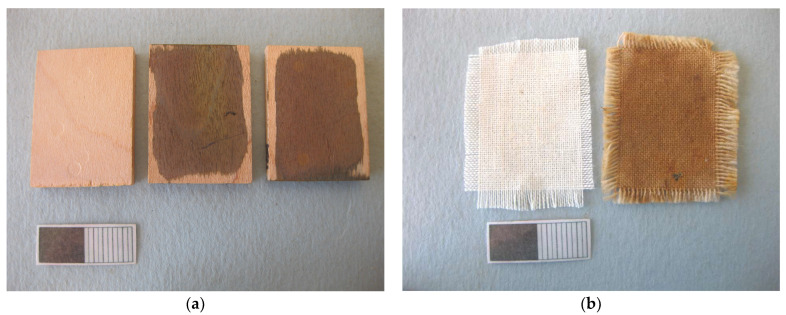
(**a**) Wooden mock-ups before staining (**left**) and after staining (**right**). (**b**) Textile mock-ups before staining (**left**) and after staining process (**right**).

For the wooden mock-ups, maple wood (*Acer platanoides* L.) was selected due to its light color, which facilitated visual monitoring during the cleaning process, and its low tannin content, which minimized interference with the artificial staining protocol. Six mock-ups measuring 30 mm in length and 22 × 5 mm in cross-section (tangential × radial) were prepared. Artificial staining was carried out by applying ferrous chloride to the tangential surfaces (Figure 18a). The amount of iron oxides and hydroxy-oxides deposited on each mock-up was estimated by measuring the oven-dry weight before and after staining, with an average increase of approximately 20 mg across 10 replicates.

For the textile mock-ups, cotton was selected due to its relatively simple chemical composition as a cellulosic fabric. This choice helped minimize the experimental variables during the cleaning process and enable a comparative assessment of cleaning methods between both substrates. The cotton fabric, supplied by Testfabrics Inc.©, West Pittston, PA, USA (catalog number 493U), was plain-weave, undyed and unbleached, with a griege construction and an approximate weight of 149 g/m^2^.

Six textile mock-ups were initially cut to 25 × 35 mm and then frayed along the edges until the intact woven area measured 20 × 30 mm, with the 30 mm side corresponding to the weft direction. Fraying ensured consistency in the dimensions of the woven portions through the study. Prior to cutting, the cotton fabric was immersed twice in slightly warm deionized water (40 °C). Iron staining of the textile mock-ups was achieved using iron wool (T/IW), which resulted in an average deposition of approximately 9 mg of iron compounds per mock-up (Figure 18b).

The staining protocols for both wood and textile mock-ups are not detailed here but will be presented in a forthcoming publication. Mössbauer and ATR-FTIR Spectroscopy identified the formation of akageneite (β-FeOOH) and maghemite (γ-Fe_2_O_3_) on both substrates, although in different proportions. In cotton mock-ups maghemite constituted approximately 82% and akageneite 18%, while in maple mock-ups it constituted 18% and 66%, respectively.

### 4.2. Cleaning Methodology

The chelators investigated in this study were ethylenediaminetetraacetic acid (EDTA) (puriss pa. ≥ 98%, Sigma Aldrich, St. Louis, MO, USA) and Desferrioxamine B (DFO-B) (Desferal^®^ by Novartis, Basel, Switzerland). Both EDTA and DFO-B are hexadentate chelators [21,96].

Although the chelators examined in this study bind iron ions in a 1:1 stoichiometry to form stable complexes [21], a lower stoichiometry of 0.5:1 was selected to facilitate the formation of rigid gels. The chelator concentration was set at 6 × 10^−2^ M, as a higher concentration, combined with the necessary buffers, resulted in high-ionic-strength solutions that affected the examined gels (prevented agarose from gelling or caused shrinkage of the nano-MWR). Chelator solutions were prepared in deionized water at pH 6.5 in order to ensure compatibility with both substrates while aligning with the dissociation constants (pK_a_) required for effective iron complexion.

More specifically, for the preparation of EDTA, 0.2 M dibasic sodium phosphate (puriss pa. ≥ 98.5–101%, Sigma Aldrich) and a few drops of 1 M NaOH were used to adjust the pH. For the DFO-B, the solution was prepared using 0.1 M succinic acid (puriss pa. ≥ 99.5%, Sigma Aldrich) and 0.2 M of Trisma Base (puriss pa. ≥ 99.9%, Sigma Aldrich).

Chelators were applied to both wood and textile substrates prepared with three gel formulations: (i) 4% *w*/*v* agarose (Type I-A, Low EEO, A0169; Sigma Aldrich) prepared at 85 °C and (ii) Nanorestore^®^ extra-dry gel with medium water retention (MWR), provided by CSGI (Research Center for Colloids and Nanoscience), Florence, Italy, both of which are rigid gels, and (iii) 2.2% *w*/*v* xanthan gum (Deosen Biochemical Ltd., Zibo, China), used as a thickening agent. The concentrations of the gel formulations were selected based on preliminary tests aimed at achieving adequate wetting of the substrates to facilitate effective complexation between the chelators and iron compounds. All gels were prepared using 2 mL of the chelator solution, a volume sufficient to clean half of the mock-ups’ surfaces and enable consistent comparison across samples.

The required amount of agarose powder was mixed with 2 mL of each chelator solution and heated to 85 °C using a magnetic stirrer until fully dissolved. The solution was then allowed to cool under stirring until it reached a semi-rigid (viscous) state to prevent penetration into the porous substrates. This state was typically achieved at approximately 45 °C, as verified with the temperature sensor of a pH meter, just prior to the gelation point (a technical sheet provided by Sigma Aldrich states that the gelation point is 37.5 °C of 1.5% *w*/*v* agarose A0169). The agarose gels were then applied to the mock-ups using a plastic spatula. The semi-rigid state was specifically chosen to facilitate controlled application on 3D cultural heritage objects.

For the nano-MWR gel application, the gel was cut into 15 × 15 mm pieces, each of which was immersed in a measured volume of chelator solution (2 mL per piece) for at least 12 h prior to application.

The chelators were combined with the required quantity of xanthan gum and stirred using a magnetic stirrer until the xanthan gum was fully dissolved. The gel formulation was then allowed to rest for at least 12 h to ensure homogeneity and to allow any entrapped air bubbles to dissipate. The gel was then applied using a plastic spatula onto a sheet of Japanese paper (3 g/m^2^, spider paper), which had been pre-positioned on the surface of the mock-ups, as a barrier layer [97,98]. The gel was left undisturbed for the full duration of the application without any mechanical agitation. This specific type of Japanese paper was selected, after preliminary testing, as it minimized gel residue while allowing the gradual release of the chelator, thereby enabling effective complexation with iron corrosion products. Prior to application, 2 mL of the gel formulation was prepared fresh for each treatment.

All gels were applied for 1 h on each of six wooden and six textile mock-ups, which were covered with a polyethylene film to prevent evaporation of the chelator solution. Each mock-up underwent eight repeated applications. This number was determined based on the outcomes of preliminary cleaning tests conducted on mock-ups of both substrate types, where fewer application cycles were found to be insufficient to effectively remove iron compounds. Following each application of the rigid hydrogels, clearance was performed by placing plain agarose gel (prepared in deionized water) or nano-MWR (also in deionized water) on the treated surface five times for 15 min [48,98]. To minimize the cost agarose was reused twice after being immersed two times in deionized water for at least 12 h, whereas nano-MWR was reused up to four times following the same rinsing procedure as that applied to agarose. In the case of the xanthan gum gel, residues and soluble iron chelator complexes were removed by a cotton swab moistened with a 50% *v/v* ethanol aqueous solution, applied until no visible compounds remained on the swab [41,42].

### 4.3. Cleaning Efficacy

#### 4.3.1. Energy-Dispersive X-Ray Spectroscopy (EDS)

Energy-Dispersive X-Ray Spectroscopy (EDS) analyses were conducted on all mock-ups prior to, during and after the cleaning procedures. Measurements were performed using a JEOL JSM 6510 LV (SEM), (Jeol Ltd., Tokyo, Japan), operated under low-vacuum mode (20–30 Pa) at an accelerating voltage of 20 kV. The instrument was fitted with an Inca X–act silicon drift detector (SDD) (Oxford Instruments, High Wycomb, UK) coupled to a PentaFET^®^ Precision spectrometer (Oxford Instruments, High Wycomb, UK).

**Figure 19:**
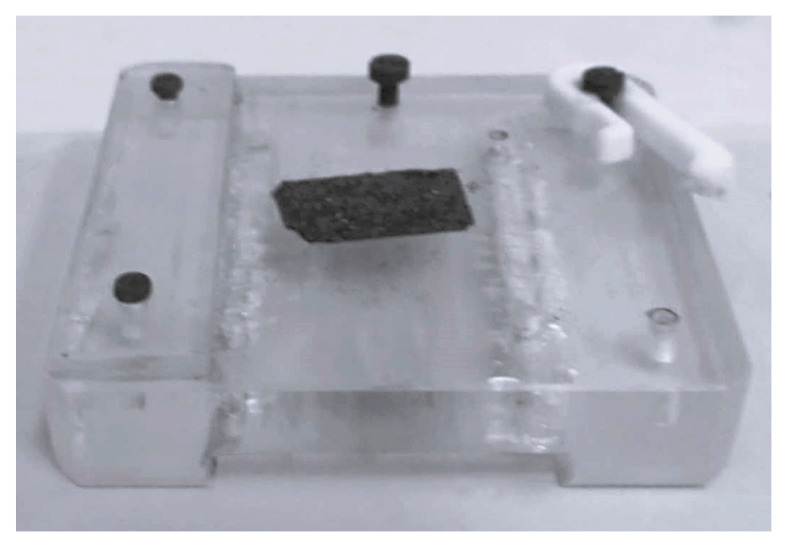
Sample holder made from Plexiglas^®^ used for placing the mock-ups during examination with SEM.

The iron (Fe) weight percentage (%wt) was calculated relative to the entire elemental profile of the organic substrate (C and O), excluding elements such as Cl and Na, which were present due to staining or the cleaning procedure, respectively. Each mock-up was placed on a custom-made Plexiglas^®^ (Polyvantis GmbH, Weiterstadt, Germany) sample holder used for the SEM analysis and was precisely positioned on it to ensure that elemental measurements targeted the identical surface area of the mock-up before, during and after the cleaning procedure (Figure 19).

The cleaning efficacy (%ΔFe) was calculated based on the difference in iron weight percentage detected before and after cleaning using Equation (1):

(1) $$\%\Delta Fe\;=\;\frac{{Fe\;}_{before\;}-\;{Fe\;}_{after}\;}{\;{Fe}_{\;before}\;\;}\;\;\;\times \;100$$

where

*Fe _before_* = %wt of Fe detected in a defined area before each chelator application;

*Fe _after_* = %wt of Fe detected in the same area after each chelator application.

#### 4.3.2. Colorimetry

Color measurements of the wooden and textile substrates were taken before and after each application using an X-Rite Lovibond portable spectrophotometer, SP60 RT series (The Tintometer Limited, Amesbury, UK). The color difference (ΔE*) was calculated according to EN 15886 [99], using Equation (2). For each measurement an average of three readings was recorded for the same area, capturing the L*, a*, b* coordinates of the CIE LAB color space.

(2) $$\Delta E*=\sqrt{{\Delta L*}^{2}+{\Delta a*}^{2}+{\Delta b*}^{2}}$$

### 4.4. Presence of Residues

#### Scanning Electron Microscopy (SEM)

To examine the surface morphology of the substrates and detect any potential gel residues on the mock-ups, Scanning Electron Microscopy (SEM) was conducted before, during and after the eighth application using a JEOL JSM–6510LV microscope, operating at an accelerating voltage of 20 kV in a low-vacuum mode (20–30 Pa). Each mock-up was placed in the SEM vacuum chamber on the sample holder, which allowed consistent positioning and the examination of the same area throughout all observations.

### 4.5. Cleaning Impact on Substrates

#### Attenuated Total Reflection–Fourier Transform Infrared Spectroscopy (ATR-FTIR)

ATR-FTIR spectra were recorded for the wooden and textile mock-ups to investigate the removal of iron corrosion products, the possible presence of gel residues and any potential impact of the cleaning methodology on both substrates. Spectra were obtained using a BRUKER ALPHA II spectrometer in ATR mode (Bruker Corporation, Billerica, MA, USA), equipped with a polycrystalline diamond crystal (RI 2.42), with a 2 mm diameter active sample area. Spectra were obtained before and after the eighth cleaning application and were recorded over the range of 4000 to 400 cm^−1^, with 24 scans per measurement, at a resolution of 4 cm^−1^.

All spectra were ATR-corrected and baseline-corrected using OPUS 8.5 SP1 software. Further processing and analysis were performed using SpectraGryph software v.1.2.17; no smoothing or additional spectrum treatments were applied. Spectra subtraction was also carried out in this software to better assess treatment effects by subtracting the spectrum of a treated mock-up from that of a reference mock-up using a subtraction factor of unity (1.0).

All spectra were normalized to unity 1 at 1035 cm^−1^, using SpectraGryph software v.1.2.17, to better highlight differences in the vibrations of the wooden and textile mock-ups, as well as the quantity of iron oxide/hydroxy-oxide in wooden mock-ups in a semi-quantitative manner, due to the suppression caused at the characteristic infrared peaks and the shoulders of the wood. This peak was chosen because it remained largely unaffected during the cleaning treatments (see the Section 2.3).

Additionally, spectra before and after artificial staining of wooden mock-ups were obtained using the Perkin Elmer Spectrum GX system with a DTGS detector (Perkin Elmer Norwalk, CT, USA) equipped with a Pike MIRacle ATR with a ZnSe crystal with a refractive index of 2.4 and a 2 mm diameter active sample area. ATR FTIR spectra were recorded in the range of 4.000 to 520 cm^−1^ (20 scans, resolution: 4 cm^−1^). For spectra recording and workup, the Spectrum v.5.3.1 software was employed. This software also offered the opportunity for spectra subtraction for better assessment of the possible changes, with a subtraction factor of unity (1.0). All acquired spectra were ATR-corrected and accordingly baseline-corrected.

### 4.6. Managing Redistribution at the Cleaning Zone Perimeter

#### Stereomicroscopy

Wooden and textile mock-ups were examined before, during and after the cleaning procedure with a stereomicroscope, (OLYMPUS SZ61) (Olympus Corporation, Tokyo, Japan) equipped with an INFINITY 1 digital camera (Lumenera Coorporation, Richmond, BC, Canada). Image capture was performed using the INFINITY CAPTURE software v6.5.3. Photographs were taken at various magnifications (0.67×, 0.80× and 1×) with a 10× eyepiece lens, enabling detailed observation of the cleaned surfaces of the mock-ups.

### 4.7. Evaluation of Gel Formulations

The performance of gel applications can be evaluated not only through measurable quantitative data but also through qualitative parameters, which can be assessed by a qualified conservator/restorer to offer a more practical understanding. This type of approach involves professional experience, knowledge and judgment [100]. This qualitative evaluation of gels was based on the following criteria: (i) preventing residue deposition, (ii) reusability, (iii) ease of application, (iv) cost, (v) suitability for use on two- and three-dimensional objects, (vi) compatibility with different chelators, and (vii) transparency and colorlessness. Each criterion was graded on a scale from 1 to 4, where 1 indicates minimal fulfillment of the criterion and 4 indicates maximal fulfillment, while 2 and 3 represent moderate and medium levels of fulfillment, respectively, as shown in Table 2.

## Figures and Tables

**Table 1:** Infrared maxima and assignments for maple wood and cotton.

Reference Maple Wood Maxima, cm^−1 a^	Assignments for Wood	Bond Vibrations	Reference Cotton Maxima, cm^−1 b^	Assignments for Cotton
3340 br	Bound water or O-H of polysaccharides	vO–H	3334 br	Bound water or O-H of cellulose
2920 m	Hemicelluloses	v_as_CH_2_	2897 m	Cellulose
2850 m	Hemicelluloses	v_s_CH_2_	2855 m	Cellulose
1735 m	Esters and acids in hemicelluloses	vC=O		
1644 br	Adsorbed water	vO–H/vC=O	1630 br	Adsorbed water
1593 m	Aromatic ring in wood lignin	vC=C aromatic skeletal vibration		
1505 m	Aromatic ring in wood lignin	vC=C aromatic		
1459 m	Lignin/carbohydrates	δCH	1454 sh	Cellulose
1424 m	Lignin/cellulose	δ(C–H)	1427 m	Cellulose (crystallinity band)
1370 m	Holocellulose	τC–H	1370 m	Cellulose
1324 m	Holocellulose	τ,wC–H	1335 m/1314 m	Cellulose
1235 s	Lignin/hemicelluloses—xylans	vC–O/ δ_ip_C–O–H	1234 w	Cellulose
1160 m	Holocellulose	v_s_C–O–C	1160 m	Cellulose
1106 sh	Holocellulose	v_as_C–O–C + *v*C–C	1108 sh	Cellulose
1053 s	Holocellulose	vC–O	1055 s	Cellulose
1035 s	Primary alcohol in holocellulose/guaiacyl COH in lignin	v(C–OH)	1032 s	Primary alcohol in cellulose
985 sh	Cellulose	δ_ip_C–C–H + δ_ip_C–C–O	985 sh	Cellulose
898 w	β-Glycosidic linkage in cellulose	νC–O–C	898 w	β-Glycosidic linkage in cellulose
877 w	α-Glycosidic linkage in hemicelluloses	νC–O–C		

^a^ [79,80,81,82,83,84], ^b^ [85,86,87,88,89,90]. Abbreviations: s: strong; m: medium; w: weak; sh: shoulder; v: stretching; v_s_: symmetric stretching; v_as_: antisymmetric stretching; δ: bending; δ_ip_: in-plane bending.

**Table 2:** Description of each grade for the different criteria.

Criteria	Description	Scale
Residue deposition	Quantity of gel residues after application	
	High/thorough clearance is needed	1
	Moderate/clearance is needed	2
	Low/clearance process is easy	3
	No gel residues after application	4
Reusability	Cannot be used again	1
	Reusable at least 2 times	2
	Reusable at least 3 times	3
	Reusable at least 4 times	4
Ease of application	Time-consuming preparation/difficulty in application	1
	Time-consuming preparation/skills demanding	2
	Quick/skills demanding/easy application	3
	Ready for application/easy application	4
Cost (supply cost in EUR)	>EUR 200	1
	>EUR 100	2
	<EUR 60	3
	<EUR 30	4
Suitability for use on two- and three-dimensional objects	2D objects only	1
	Slightly curved surfaces/weight is needed	2
	3D objects without full contact	3
	3D objects	4
Compatibility with different chelators	Not compatible	1
	Selectively compatible	2
	Generally compatible	3
	Compatible	4
Transparency and colorlessness	Not transparent/color changes not visible	1
	Semitransparent/difficult to distinguish color changes	2
	Transparent/difficult to distinguish color changes	3
	Transparent/color changes visible	4

## Data Availability

The data presented in this study are available on request from the corresponding author.
